# Correcting for selection bias in two-stage trials with multiple correlated outcomes: application to seamless phase ii/iii clinical trials

**DOI:** 10.1186/1745-6215-16-S2-P145

**Published:** 2015-11-16

**Authors:** David Robertson, A Toby Prevost, Jack Bowden

**Affiliations:** 1 MRC Biostatistics Unit, Cambridge, UK; 2 King's College, London, UK

## 

The problem of selection bias has long been recognised in the analysis of two-stage trials, where promising candidates are selected in stage 1 for confirmatory analysis in stage 2. To efficiently correct for bias, uniformly minimum variance conditionally unbiased estimators (UMVCUEs) have been proposed for a wide variety of trial settings, but where the populations are assumed to be independent. We relax this assumption and derive the UMCVUE in the multivariate normal setting with an arbitrary known covariance structure.

We explore the application of our estimator to two-stage adaptive seamless phase II/III clinical trials. In stage 1, multiple experimental treatments are simultaneously compared with a control. The one that shows the highest standardised treatment difference is then selected for confirmatory analysis in stage. Our UMVCUE allows for unequal number of subjects allocated to each treatment in stage 1, and different (known) treatment variances. We demonstrate the use of our estimator in simulation studies, and show the gain in efficiency over just using the stage 2 data.

